# Current Insights into Pasireotide Therapy for Uncontrolled Acromegaly: Biochemical Response, Tumor Reduction, and Glycemic Safety in a Real-World Latin American Cohort

**DOI:** 10.3390/life16071114

**Published:** 2026-07-03

**Authors:** Alin Abreu Lomba, David Alexander Vernaza Trujillo, Carlos Andrés Tafur Monje, Wilfredo Antonio Rivera-Martínez, Cesar Augusto Mejía Vélez, Juan S. Izquierdo-Condoy

**Affiliations:** 1 Grupo Interinstitucional de Medicina Interna (GIMI1), Universidad Libre, Cali 760045, Colombia; abreu.lomba@gmail.com (A.A.L.); vernaza4@hotmail.com (D.A.V.T.);; 2 Endocrinology Department, Clínica Imbanaco, Cali 760042, Colombia; 3 Department of Public Health, Pontificia Universidad Javeriana, Cali 760031, Colombia; 4 Clinical Research Center, Clínica Imbanaco, Cali 760042, Colombia; 5 Endocrinology Department, Clínica Nueva de Cali, Cali 760044, Colombia; 6 Endocrinology Department, IPS Nauru, Cali 760042, Colombia; antonioriveramartinez@gmail.com; 7 One Health Research Group, Universidad de Las Américas, Quito 170513, Ecuador

**Keywords:** acromegaly, pasireotide, Individualized endocrine care, real-world evidence, metabolic safety

## Abstract

Background/Objectives: Acromegaly is a chronic endocrine disorder caused mainly by GH-secreting pituitary adenomas, leading to excess GH and elevated IGF-1. Although surgery is first-line therapy, many patients require medical treatment, and remission is often not achieved with first-generation somatostatin receptor ligands (SRLs). Pasireotide, a second-generation SRL, offers superior biochemical and tumor control but is associated with hyperglycemia. This study aimed to evaluate real-world outcomes associated with pasireotide treatment in patients with acromegaly inadequately controlled on first-generation SRLs, with IGF-1 normalization as the primary endpoint. Secondary outcomes included GH control, tumor response, and glycemic safety. Methods: We conducted a historical cohort study of adults with acromegaly treated at Clínica Imbanaco (Cali, Colombia) between 2017 and 2024. Eligible patients had residual tumors and persistently elevated GH and/or IGF-1 levels above the age-adjusted upper limit of normal despite treatment with clinically adequate doses of first-generation SRLs, as well as 12 months of continuous pasireotide treatment and follow-up after pasireotide initiation. Demographic, biochemical, imaging, and glycemic data were collected. Statistical analysis included paired and independent Student’s *t*-tests, Wilcoxon signed-rank tests, McNemar’s test, and Fisher’s exact test, with significance set at *p* < 0.05. Results: Fourteen patients (50% female; mean age 52.1 ± 14.5 years) were included. After 12 months, mean IGF-1 decreased from 2.73 ± 0.73 to 0.99 ± 0.56 × ULN, and 50% achieved IGF-1 normalization. Additionally, 35.7% achieved GH < 1 ng/mL, and 14.3% achieved combined control. Mean tumor diameter decreased by −3.26 mm (95% CI −4.56 to −1.95; *p* < 0.001). HbA1c increased from 5.56% to 6.05%, while type 2 diabetes mellitus prevalence rose from 14.3% to 35.7%. No patient discontinued pasireotide due to metabolic adverse events. Conclusions: Pasireotide was associated with favorable biochemical and tumor responses in patients with acromegaly inadequately controlled on first-generation SRLs under real-world conditions. Although treatment was associated with higher HbA1c and increased diabetes incidence, proactive monitoring and early management of hyperglycemia may have supported treatment persistence.

## 1. Introduction

Acromegaly is a chronic, progressive endocrine disorder characterized by growth hormone (GH) hypersecretion and the consequent elevation of insulin-like growth factor 1 (IGF-1). In the vast majority of cases, etiology corresponds to a GH-secreting pituitary adenoma, accounting for up to 98% of diagnoses [1]. This condition results in multiple somatic, metabolic, and cardiovascular complications, with a negative impact on both quality of life and survival, particularly when adequate biochemical control is not achieved [2,3].

Despite advances in diagnosis and treatment, acromegaly remains an underdiagnosed and undertreated disease. The average interval between symptom onset and definitive diagnosis may exceed 6 to 8 years [1,2,3]. In Colombia, prevalence has not been clearly established; however, recent registries such as RAPACO (Registro de Pacientes con Acromegalia en Colombia; Colombian Acromegaly Patient Registry) report a mean age of approximately 49 years, female predominance, and a high frequency of macroadenomas [3]. This diagnostic delay is associated with an increased burden of comorbidities, including arterial hypertension, cardiac dysfunction, sleep apnea, glucose abnormalities, and osteoarticular complications [1,2,3,4,5,6,7].

The primary therapeutic goal is to achieve sustained biochemical control—defined as IGF-1 levels within the age-adjusted normal range—in accordance with the recommendations of the 14th Acromegaly Consensus Conference, along with tumor volume reduction and clinical improvement [8,9]. Although transsphenoidal surgery remains the first-line treatment, a considerable proportion of patients require complementary medical therapy with first-generation somatostatin analogues, dopamine agonists, or GH receptor antagonists [1,2,3,4]. In this setting, pasireotide, a second-generation somatostatin analogue, has shown higher biochemical and tumor response rates in selected cases compared with conventional treatments, although its use is associated with an increased risk of hyperglycemia [5,6,7].

In this context, the present study aimed to describe biochemical response, tumor outcomes, and glycemic changes associated with pasireotide treatment in patients with acromegaly inadequately controlled on first-generation somatostatin receptor ligands in a real-world clinical setting.

## 2. Materials and Methods

### 2.1. Study Design and Setting

We conducted a historical cohort study that included patients with acromegaly treated between 2017 and 2024 at Clínica Imbanaco, a private referral medical center in Cali, Colombia.

### 2.2. Population and Eligibility Criteria

The cohort consisted of adult patients with a confirmed diagnosis of acromegaly. All patients, except one, had undergone transsphenoidal surgery and had previously received medical therapy with first-generation somatostatin receptor ligands (SRLs). Eligible patients were required to have initiated pasireotide LAR and to remain on continuous treatment throughout the 12-month follow-up period. All included patients completed scheduled follow-up assessments while receiving ongoing pasireotide therapy. Patients were eligible if they met the following criteria: (i) persistently elevated IGF-1 levels above the age-adjusted upper limit of normal (ULN) and/or elevated random GH levels despite at least 3–6 months of treatment with clinically adequate doses of first-generation SRLs (lanreotide 120 mg every 4 weeks or octreotide LAR 30–40 mg every 4 weeks), as determined by the treating endocrinologist; (ii) residual tumor on imaging; (iii) a minimum follow-up of 12 months after initiation of pasireotide. Duration of medical treatment before first-generation SRLs refers specifically to acromegaly-directed pharmacological therapy (e.g., dopamine agonists) administered prior to initiation of first-generation somatostatin receptor ligands and does not include treatment for comorbid conditions.

Pasireotide LAR was initiated at 20 mg every 28 days in all patients and subsequently titrated to 40 mg or 60 mg according to biochemical response and clinical judgment during follow-up. Patients were typically evaluated every 3–6 months. Dose escalation was considered when IGF-1 levels remained above the age-adjusted ULN after at least 3 months at a stable dose.

All GH and IGF-1 measurements were performed in the same institutional laboratory using chemiluminescent immunoassays, with consistent assay platforms and age-adjusted reference intervals throughout the study period.

Patients with incomplete medical records or comorbidities that could interfere with outcome assessment, such as uncontrolled type 2 diabetes mellitus (T2DM) or significant liver disease, were excluded. During the study period, no patients received pegvisomant or combination medical therapy, and none underwent pituitary radiotherapy prior to pasireotide initiation. No patient received pituitary radiotherapy during the 12-month follow-up period.

#### 2.2.1. Outcome Definitions

− Biochemical control: defined as IGF-1 levels within the age-adjusted normal range (≤1.0 × upper limit of normal [ULN]). IGF-1 values were additionally expressed as multiples of the age-adjusted ULN using institutional laboratory reference intervals for somatomedin C (IGF-1). As an additional endpoint, absolute disease control was defined as simultaneous normalization of IGF-1 (≤1.0 × ULN) and random GH < 1 ng/mL.

In accordance with recent international consensus recommendations, normalization of age-adjusted IGF-1 was considered the primary biochemical endpoint, whereas random GH was evaluated as a complementary marker of disease control. GH suppression during oral glucose tolerance testing (OGTT), including the <0.4 ng/mL threshold, was not systematically available because of the retrospective design and therefore was not incorporated into the definition of biochemical control [8,9].

− Tumor size: assessed by magnetic resonance imaging (MRI), considering the reduction in the maximum adenoma diameter.
− Glycemic profile: included fasting plasma glucose, serial HbA1c measurements, and evaluation of T2DM onset or progression during follow-up. Baseline glycemic measurements were obtained immediately prior to pasireotide initiation and reflect the patient’s metabolic status at the time of switching from first-generation SRLs. Glycemic status was primarily categorized using HbA1c according to American Diabetes Association criteria as normal glycemia (HbA1c < 5.7%), prediabetes (HbA1c 5.7–6.4%), and diabetes mellitus (HbA1c ≥ 6.5%). Fasting plasma glucose was also recorded during routine follow-up and interpreted according to ADA thresholds: normal fasting glucose <100 mg/dL, impaired fasting glucose 100–125 mg/dL, and diabetes-range fasting glucose ≥126 mg/dL. HbA1c was selected as the primary glycemic outcome because it was consistently available across all study visits and provided a standardized measure of longitudinal glycemic control [10].

#### 2.2.2. Follow-Up

All patients were monitored for at least 12 months after pasireotide initiation. Patients were typically evaluated every 3–6 months. At baseline (before the first pasireotide injection) and at each follow-up visit, biochemical parameters (GH and IGF-1), metabolic variables (fasting plasma glucose, HbA1c, and T2DM status), and radiological findings (MRI) were recorded. Follow-up assessments at 6 and 12 months were performed under a stable pasireotide dose for at least 3 months. This was not an eligibility criterion but rather a characteristic of the scheduled follow-up assessments.

### 2.3. Statistical Analysis

Categorical variables were described as absolute and relative frequencies. For quantitative variables, distribution was assessed using the Shapiro–Wilk test. Normally distributed variables were expressed as mean ± standard deviation (SD). Longitudinal within-patient comparisons (baseline vs. follow-up) for GH, IGF-1 (both absolute values and multiples of the age-adjusted upper limit of normal [ULN]), tumor diameter, and HbA1c were performed using paired Student’s *t*-tests. As a sensitivity analysis, non-parametric Wilcoxon signed-rank tests were additionally performed for key longitudinal outcomes. Comparisons between independent subgroups according to pasireotide dose or prior first-generation somatostatin receptor ligand type were performed using independent-samples Student’s *t*-tests and were considered exploratory. Categorical paired variables were analyzed using McNemar’s test when applicable, whereas categorical comparisons between independent groups were analyzed using Fisher’s exact test or the Chi-square test, as appropriate. A *p*-value < 0.05 was considered statistically significant. All analyses were performed using IBM SPSS Statistics for Windows, version 29.0 (IBM Corp., Chicago, IL, USA).

### 2.4. Ethical Statement

This study was conducted in full compliance with national regulations and in accordance with the principles outlined in the Declaration of Helsinki. The study protocol received ethical approval from the Health Research Ethics Committee of Clínica Imbanaco (approval code CEI-878). The requirement for informed consent was waived by the Ethics Committee because of the retrospective nature of the study and the use of anonymized clinical data.

## 3. Results

### 3.1. Baseline Cohort Characteristics

**Figure 1:**
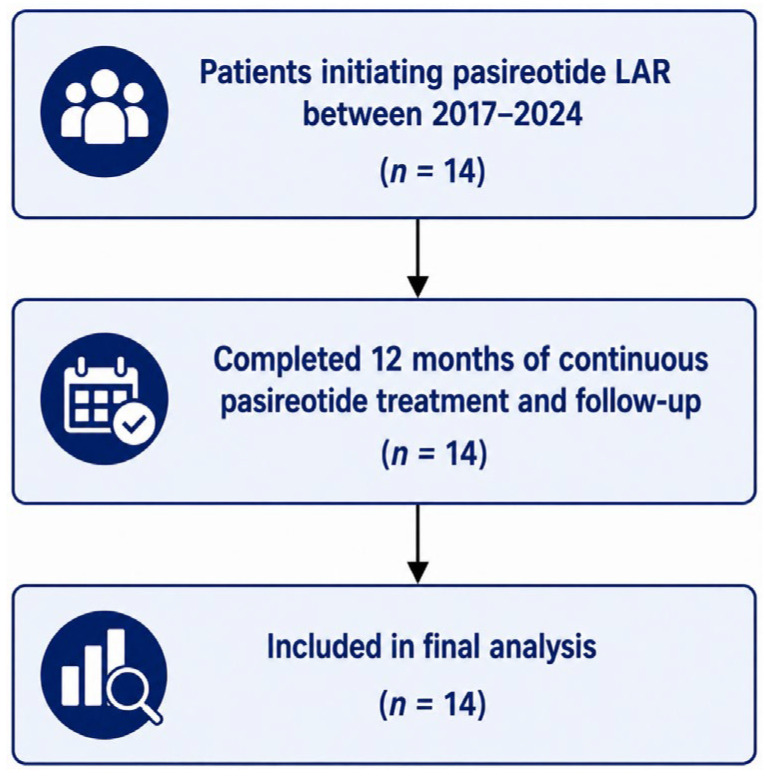
Patient disposition flow diagram. Fourteen patients initiated pasireotide LAR between 2017 and 2024. All remained on continuous treatment throughout the 12-month follow-up, completed scheduled clinical assessments, and were included in the final analysis. No patient was excluded because of incomplete medical records, uncontrolled T2DM, significant liver disease, early discontinuation, loss to follow-up, pituitary radiotherapy during follow-up, or adverse events.

Fourteen consecutive patients initiated pasireotide LAR during the study period. All 14 were screened, met the inclusion criteria, remained on continuous pasireotide treatment throughout the 12-month follow-up period, completed scheduled follow-up assessments, and were included in the final analysis. No patients were excluded because of incomplete medical records, uncontrolled T2DM, significant liver disease, early discontinuation, loss to follow-up, pituitary radiotherapy during follow-up, or adverse events. All patients remained on pasireotide throughout the 12-month observation period (Figure 1).

A total of 14 patients with acromegaly were included, of whom 7 (50%) were male. Mean age was 52.1 ± 14.5 years, and mean time since diagnosis was 6.6 ± 3.2 years. Thirteen patients (92.9%) had undergone prior pituitary surgery, and all had residual tumor at pasireotide initiation. The mean residual tumor diameter measured immediately before pasireotide initiation was 10.6 ± 3.5 mm. Two patients (14.3%) had a known diagnosis of type 2 diabetes mellitus (T2DM) at baseline (Table 1).

### 3.2. Previous Treatment and Transition to Pasireotide

Before starting pasireotide, 10 patients (71.4%) had received lanreotide and 4 (28.6%) octreotide. The mean duration of first-generation SRL treatment was 10.9 ± 3.6 months for lanreotide and 9.5 ± 3.9 months for octreotide (*p* = 0.529). Tumor size before initiation of first-generation SRLs was similar between the lanreotide and octreotide groups (11.7 ± 2.9 mm vs. 9.8 ± 2.5 mm; *p* = 0.273). Tumor size changes during first-generation SRL therapy before pasireotide were small in both groups (1.3 ± 2.1 mm vs. 1.8 ± 2.1 mm; *p* = 0.176) (Table 2).

### 3.3. Pasireotide Dosing During Follow-Up

All patients received pasireotide for at least 12 months. The initial mean dose was 20 mg and was titrated according to clinical and biochemical response to a final mean dose of 42.9 ± 13.3 mg. At 12 months, 2 patients (14.3%) were on 20 mg, 8 (57.1%) on 40 mg, and 4 (28.6%) on 60 mg.

### 3.4. Biochemical Response

At baseline, no patient met biochemical control. Mean GH and IGF-1 levels were 6.36 ± 3.21 ng/mL and 618.71 ± 224.26 ng/mL, respectively. When stratified by final pasireotide dose (≤40 mg vs. >40 mg), exploratory comparisons showed that patients who ultimately required >40 mg had higher baseline GH (9.53 ± 3.42 vs. 5.10 ± 2.17 ng/mL; *p* = 0.01) and higher IGF-1 (823.00 ± 292.41 vs. 537.00 ± 134.81 ng/mL; *p* = 0.01), as well as a larger baseline tumor diameter (13.75 ± 4.79 vs. 9.40 ± 2.12 mm; *p* = 0.03) (Table 1 and Appendix A, Table A1).

After 12 months of pasireotide treatment, significant within-patient biochemical and tumor improvements were observed (Table 3). Mean IGF-1 decreased from 618.7 ± 224.3 ng/mL at baseline to 226.1 ± 136.5 ng/mL at 12 months (mean difference −392.6 ng/mL; 95% CI −502.1 to −283.0; paired *t*-test, *p* < 0.001). When expressed as multiples of the age-adjusted upper limit of normal (ULN), mean IGF-1 decreased from 2.73 ± 0.73 × ULN at baseline to 0.99 ± 0.56 × ULN at 12 months (mean difference −1.74 × ULN; 95% CI −2.19 to −1.29; *p* < 0.001). Biochemical normalization (IGF-1 ≤ 1.0 × ULN) was achieved in 50% of patients. Mean GH levels declined from 6.36 ± 3.21 ng/mL to 2.00 ± 1.22 ng/mL (mean difference −4.36 ng/mL; 95% CI −5.84 to −2.89; *p* < 0.001). Tumor diameter was significantly reduced from 10.64 ± 3.54 mm at baseline to 7.39 ± 2.95 mm at 12 months (mean reduction −3.26 mm; 95% CI −4.56 to −1.95; *p* < 0.001). Categorically, 35.7% of patients achieved GH < 1 ng/mL, 50.0% achieved age-adjusted IGF-1 normalization, and 14.3% achieved simultaneous biochemical control.

Non-parametric Wilcoxon signed-rank tests yielded similar results for IGF-1 (absolute values and ×ULN), GH, and tumor diameter (all *p* ≤ 0.001).

Exploratory comparisons between dose subgroups showed differences in biochemical and tumor-related measures. At 12 months, patients in the >40 mg group maintained higher absolute GH levels (3.00 ± 1.49 vs. 1.60 ± 0.89 ng/mL; *p* = 0.05) and IGF-1 levels (344.50 ± 121.20 vs. 178.81 ± 115.31 ng/mL; *p* = 0.03) than those in the ≤40 mg group. Findings were consistent when IGF-1 was expressed as multiples of the age-adjusted ULN (Table A1). Nevertheless, the magnitude of hormonal reduction was greater in the higher-dose group. Specifically, the decrease in GH from baseline was −6.53 ± 2.88 ng/mL in the >40 mg group, significantly greater than the −3.50 ± 1.93 ng/mL observed in the ≤40 mg group (*p* = 0.04). In the case of IGF-1, the absolute reduction was larger in the >40 mg group, albeit without reaching statistical significance (−478.50 ± 237.93 vs. −358.19 ± 168.96 ng/mL; *p* = 0.30). Regarding tumor evolution, the final diameter at 12 months remained larger in the >40 mg group (10.00 ± 2.16 vs. 5.53 ± 2.93 mm; *p* = 0.02), although the absolute reduction was comparable between subgroups (−3.75 ± 2.63 vs. −3.87 ± 3.37 mm; *p* = 0.95) (Table 3 and Table A1).

When analyzing categorical variables, no statistically significant differences were observed between dose categories (≤40 mg vs. >40 mg) in terms of GH control, IGF-1 control, or tumor shrinkage ≥20%.

Dose-stratified comparisons are presented as exploratory analyses in Table A1.

### 3.5. Glycemic Profile

**Figure 2:**
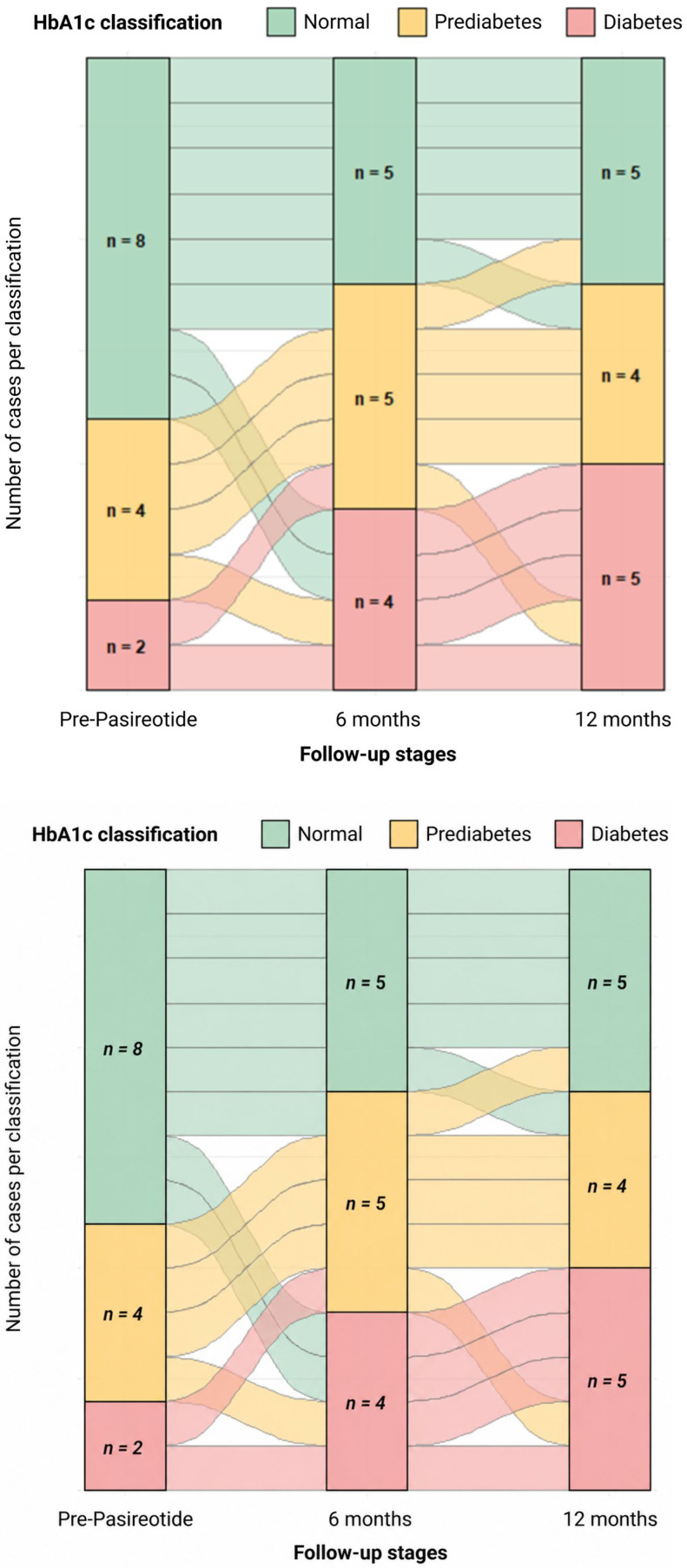
Transitions in glycemic status according to HbA1c categories at baseline (pre-pasireotide), 6 months, and 12 months after pasireotide initiation.

Mean HbA1c showed an early increase followed by stabilization. It rose from 5.56% at baseline to 6.31% at 6 months and then decreased slightly to 6.05% at 12 months (Figure 2). In parallel, the proportion of patients with type 2 diabetes mellitus (T2DM) increased progressively from 2 cases (14.3%) at baseline to 4 cases (28.6%) at 6 months and 5 cases (35.7%) at the end of the 12-month follow-up (Table 3). No clinically significant non-glycemic adverse events leading to treatment discontinuation were observed during follow-up.

Wilcoxon testing confirmed a significant increase in HbA1c between baseline and 6 months (*p* = 0.003), whereas no significant change was observed between 6 and 12 months (*p* = 0.102).

Patients were classified as normal, prediabetes, or diabetes according to HbA1c thresholds. The diagram illustrates shifts in glycemic classification over time. Management of treatment-emergent hyperglycemia followed published expert consensus recommendations. Created with BioRender.com.

## 4. Discussion

This study evaluated real-world biochemical, tumor, and metabolic outcomes associated with pasireotide treatment in patients with acromegaly inadequately controlled on first-generation somatostatin receptor ligands (SRLs) in a real-world clinical practice setting. Acromegaly represents a clinical challenge due to its low prevalence, delayed diagnosis, and the limited access to effective therapies in real-life scenarios. In Colombia, recent registries have reported that up to 78% of cases correspond to macroadenomas, and that biochemical control is achieved in only a minority of patients treated with conventional medical therapy [3,4,5]. This situation translates into a higher burden of comorbidities and increased use of healthcare resources [5]. Against this backdrop, the availability of drugs such as pasireotide, which may increase the likelihood of biochemical control in selected patients, becomes particularly relevant.

In our cohort, composed mostly of patients previously treated surgically with residual tumor, the use of pasireotide was associated with significant reductions in GH and IGF-1, achieving partial normalization in approximately half of the cases and complete control in 14.3%. These findings are consistent with those reported in national series and with results from pivotal trials, such as the study by Colao et al. (2014) and PAOLA (2014), where pasireotide showed higher biochemical response rates than first-generation SSAs in selected patients with inadequate biochemical control [6,7]. The high failure rate of first-generation SSAs, which can reach 70% [2], further underscores pasireotide’s role as an important second-line option.

Importantly, exposure to first-generation SRLs was comparable between patients previously treated with lanreotide and those treated with octreotide, with no significant differences in prior treatment duration or pre-switch tumor dynamics. The only significant difference was a larger baseline tumor size among patients initially managed with lanreotide, which is consistent with real-world clinical decision-making, where tumor burden often guides the choice of SRLs. This overall homogeneity supports the classification of all patients as inadequately controlled despite clinically adequate exposure to first-generation SRLs prior to pasireotide initiation, as biochemical activity persisted under optimized first-line therapy.

It should be emphasized that differences in remission criteria across studies hinder direct comparison. Whereas earlier clinical trials accepted GH ≤ 2.5 µg/L and/or IGF-1 < 1.3 times the age-adjusted upper limit of normal (ULN), current recommendations define strict IGF-1 normalization as the central parameter, with random GH < 1.0 µg/L only as a supportive criterion [1,8,9]. In our cohort, applying these more stringent criteria reduced the proportion of patients classified as being in remission, providing a more rigorous assessment of disease activity and its potential clinical implications.

Several real-world studies reinforce this perspective. Stelmachowska-Banaś et al. (2022) reported biochemical control rates exceeding 60% in a Polish cohort during the first 12 months of follow-up [11]. Urbani et al. (2024) showed that, in the long term, the prevalence of diabetes increased from 33% to 54%, with a discontinuation rate of 36% due to inefficacy or adverse events [12]. Chiloiro et al. (2024) explored the influence of GH receptor polymorphisms on response to second-line therapies, suggesting that genetic characterization could optimize patient selection [13]. Finally, the meta-analysis by the Spanish group led by Biagetti et al. (2025), which included 590 patients, estimated an overall biochemical control rate of 26.5% at 12 months, with IGF-1 and GH normalization achieved in 36.3% and 34.8%, respectively [14]. These findings support the clinical utility of pasireotide, although with variability influenced by methodological differences and heterogeneous response definitions. Although our estimates are directionally consistent with these larger cohorts, the confidence intervals observed in our study were wider because of the substantially smaller sample size and should therefore be interpreted with appropriate caution.

Regarding tumor volume, our series documented clinically relevant tumor shrinkage in a substantial proportion of patients, consistent with previous reports suggesting an antiproliferative effect of pasireotide in addition to its antisecretory action [15,16]. This finding is particularly relevant in the context of residual tumor after surgery.

The metabolic profile remains one of the main challenges associated with treatment. In our cohort, the prevalence of T2DM increased from 14.3% to 35.7% during follow-up, accompanied by higher HbA1c levels. Baseline glycemic values were obtained immediately before pasireotide initiation and therefore reflect the metabolic state after exposure to first-generation SSAs rather than untreated glycemic status. As FG-SSAs may worsen glucose metabolism in a subset of patients, this aspect should be considered when interpreting subsequent changes in glucose control under pasireotide. This pattern aligns with the literature, where up to 56% of patients treated with pasireotide experience glycemic alterations [17,18,19,20,21]. Unlike first-generation SRLs, whose glycemic impact is limited, pasireotide induces hyperglycemia due to its high affinity for SSTR5 in pancreatic β-cells [18]. However, in contrast to other series in which up to one-third of patients discontinued treatment due to metabolic deterioration [12], no patient in our cohort required treatment withdrawal. This finding may be related to early and personalized management of dysglycemia, in line with national expert recommendations, which may have contributed to maintaining acceptable glycemic control [22,23,24,25,26].

Taken together, these findings suggest that pasireotide treatment was associated with favorable biochemical and tumor outcomes in patients with acromegaly inadequately controlled on first-generation SRLs under real-world conditions, while highlighting the need for strict monitoring of glucose metabolism. The adoption of current IGF-1–centered criteria provides a more rigorous assessment of treatment response, although it reduces the proportion of patients considered in remission compared with earlier definitions [1,8,9]. These observations underscore the value of local real-world data and suggest that proactive glucose management may favor therapeutic persistence in patients treated with pasireotide.

### Limitations

This study has several limitations that should be considered when interpreting the results. First, it was a retrospective study with a small sample size and no comparator group, which restricts the ability to establish causal associations and limits the generalizability of the findings. Second, the follow-up was limited to 12 months, precluding assessment of the long-term sustainability of the observed effects and the evaluation of hard clinical outcomes such as mortality or sustained tumor progression. Third, being a single-center study, the results reflect the experience of a single clinical setting and may not be extrapolable to other healthcare contexts with different access to therapies or population characteristics. The economic impact and cost-effectiveness of pasireotide were not evaluated and may influence treatment accessibility in resource-constrained settings.

Additionally, although all biochemical measurements were performed in the same institutional laboratory using consistent assay platforms, manufacturer-level calibration details were not systematically retrievable due to the retrospective design; therefore, manufacturer-specific calibration differences cannot be excluded. GH suppression during oral glucose tolerance testing (OGTT), including OGTT-derived nadir GH values, was not systematically available, which prevented assessment of biochemical control using OGTT-based GH suppression criteria. Detailed longitudinal information on specific antidiabetic agents initiated, discontinued, or adjusted during follow-up was not systematically available for all patients; therefore, agent-specific glycemic management and comparisons between glucose-lowering strategies could not be analyzed.

SSTR2 receptor expression was not systematically available and therefore could not be evaluated as a predictor of treatment response. Similarly, genetic or molecular factors that could influence treatment response were not systematically explored, nor was the impact on quality-of-life outcomes assessed, both of which represent relevant dimensions in the comprehensive evaluation of therapeutic response. Representative MRI images suitable for publication were not systematically available for all patients; therefore, tumor response was assessed quantitatively using standardized MRI measurements rather than illustrative imaging examples.

In addition, the small sample size limits statistical power, particularly for subgroup analyses, which should be interpreted as exploratory and hypothesis-generating. The relatively wide confidence intervals observed for some outcomes, particularly IGF-1–related measures, reflect the limited sample size and should be considered when interpreting the precision of the estimates. Because of the observational design and absence of a comparator group, the study cannot establish causal treatment effects; therefore, findings should be interpreted as associations observed in routine clinical practice.

## 5. Conclusions

Pasireotide was associated with favorable biochemical responses, assessed using contemporary IGF-1–centered remission criteria, along with clinically relevant tumor size reduction in patients with acromegaly inadequately controlled on first-generation somatostatin receptor ligands. Although glycemic parameters worsened in a subset of patients, no treatment discontinuations occurred, suggesting that proactive metabolic monitoring may support therapeutic persistence in real-world clinical practice. Larger prospective, multicenter studies are warranted to confirm these findings and to better define the long-term safety and durability of response.

## Figures and Tables

**Table 1:** Baseline clinical and biochemical characteristics of the cohort.

Variable	Value
Sex	
Male	7 (50.0%)
Female	7 (50.0%)
Age (years)	52.1 ± 14.5
Clinical history	
Time since diagnosis (years)	6.6 ± 3.2
Previous pituitary surgery	13 (92.9%)
Type 2 diabetes mellitus	2 (14.3%)
Previous treatment	
Duration of medical treatment before first-generation SRLs (months)	12.1 ± 3.5
Duration of first-generation SRLs treatment (months)	10.4 ± 5.4
Pasireotide treatment	
Initial dose (mg)	20.0 ± 0.0
Final dose (mg)	42.9 ± 13.3
Biochemical profile at baseline	
HbA1c (%)	5.6 ± 0.6
GH (ng/mL)	6.4 ± 3.2
Patients with controlled GH	0 (0.0%)
IGF-1 (ng/mL)	618.7 ± 224.3
Patients with controlled IGF-1	0 (0.0%)
Tumor characteristics	
Tumor diameter (mm)	10.6 ± 3.5

Duration of medical treatment before first-generation SRLs refers to acromegaly-directed therapy only (e.g., dopamine agonists). Tumor diameter corresponds to the maximum residual adenoma diameter measured by MRI immediately before pasireotide initiation.

**Table 2:** Prior first-generation SRL treatment and tumor characteristics.

	Lanreotide (*n* = 10)		Octreotide (*n* = 4)		*p*-Value
	Mean	±SD	Mean	±SD	
Duration of medical treatment before first-generation SRLs (months)	10.2	6.1	9.8	2.5	0.872
Tumor change before pasireotide (mm)	1.3	2.1	1.8	2.1	0.176
Tumor size before first-generation SRLs (mm)	11.7	2.9	9.8	2.5	0.273
Duration of first-generation SRLs treatment (months)	10.9	3.6	9.5	3.9	0.529

Duration of medical treatment before first-generation SRLs refers to acromegaly-directed therapy only (e.g., dopamine agonists).

**Table 3:** Biochemical and tumor outcomes at 12 months.

Outcome	Baseline Mean ± SD	Follow-Up Mean ± SD	Mean Difference (Δ)	95% CI of Δ	*p*-Value
IGF-1 (ng/mL)	618.7 ± 224.3	226.1 ± 136.5	−392.6	−502.1 to −283.0	<0.001
IGF-1 (×ULN)	2.73 ± 0.73	0.99 ± 0.56	−1.74	−2.19 to −1.29	<0.001
GH (ng/mL)	6.36 ± 3.21	2.00 ± 1.22	−4.36	−5.84 to −2.89	<0.001
Tumor diameter (mm)	10.64 ± 3.54	7.39 ± 2.95	−3.26	−4.56 to −1.95	<0.001
HbA1c (%)—baseline to 6 months	5.56 ± 0.63	6.31 ± 0.96	+0.75	+0.30 to +1.20	0.003
HbA1c (%)—6 to 12 months	6.31 ± 0.96	6.05 ± 0.60	−0.26	−0.55 to +0.03	0.07

## Data Availability

The original contributions presented in this study are included in the article. Further inquiries can be directed to the corresponding author.

## Appendix A

**Table A1:** Exploratory dose-stratified comparisons according to final pasireotide dose (≤40 mg vs. >40 mg).

	Pasireotide ≤ 40 mg (*n* = 10)		Pasireotide > 40 mg (*n* = 4)		*p*-Value
	Mean	±SD	Mean	±SD	
**Glycemic profile**					
HbA1c prior to pasireotide (%)	5.6	0.5	5.4	0.9	0.622
HbA1c at 6 months (%)	6.6	1.0	5.7	0.5	0.134
HbA1c at 12 months (%)	6.1	0.6	6.0	0.6	0.781
**Biochemical response**					
IGF-1 prior to pasireotide (ng/mL)	537.0	134.8	823.0	292.4	0.024
IGF-1 at 12 months (ng/mL)	178.8	115.3	344.5	121.2	0.034
IGF-1 change at 12 months (ng/mL)	358.2	169.0	478.5	237.9	0.302
IGF-1 baseline (×ULN)	2.41	0.46	3.55	0.63	0.006
IGF-1 at 12 months (×ULN)	0.78	0.35	1.54	0.64	0.020
IGF-1 change (×ULN)	−1.63	0.51	−2.01	0.59	0.29
GH prior to pasireotide (ng/mL)	5.1	2.2	9.5	3.4	0.012
GH at 12 months (ng/mL)	1.6	0.9	3.0	1.5	0.047
GH change at 12 months (ng/mL)	3.5	1.9	6.5	2.9	0.039
**Tumor outcomes**					
Maximal tumor diameter prior to pasireotide (mm)	9.4	2.1	13.8	4.8	0.031
Maximal tumor diameter at 12 months (mm)	5.5	2.9	10.0	2.2	0.018
Change in maximal tumor diameter (mm)	3.9	3.4	3.8	2.6	0.951
